# Simulated Annealing Based Algorithm for Identifying Mutated Driver Pathways in Cancer

**DOI:** 10.1155/2014/375980

**Published:** 2014-05-26

**Authors:** Hai-Tao Li, Yu-Lang Zhang, Chun-Hou Zheng, Hong-Qiang Wang

**Affiliations:** ^1^College of Information and Communication Technology, Qufu Normal University, Rizhao 276826, China; ^2^College of Jia Sixie Agriculture, Weifang University of Science and Technology, Shouguang 262700, China; ^3^College of Electrical Engineering and Automation, Anhui University, Hefei 230000, China; ^4^Intelligent Computing Lab, Hefei Institute of Intelligent Machines, Chinese Academy of Sciences, Hefei 230000, China

## Abstract

With the development of next-generation DNA sequencing technologies, large-scale cancer genomics projects can be implemented to help researchers to identify driver genes, driver mutations, and driver pathways, which promote cancer proliferation in large numbers of cancer patients. Hence, one of the remaining challenges is to distinguish functional mutations vital for cancer development, and filter out the unfunctional and random “passenger mutations.” In this study, we introduce a modified method to solve the so-called maximum weight submatrix problem which is used to identify mutated driver pathways in cancer. The problem is based on two combinatorial properties, that is, coverage and exclusivity. Particularly, we enhance an integrative model which combines gene mutation and expression data. The experimental results on simulated data show that, compared with the other methods, our method is more efficient. Finally, we apply the proposed method on two real biological datasets. The results show that our proposed method is also applicable in real practice.

## 1. Introduction

Cancer is a fatal disease which is extremely complex. Researchers have found that cancer should be arisen by single-nucleotide mutations, larger copy-number aberrations, or structural aberrations [1]. The dreadful feature of cancer cells is infinite proliferation. These abnormal cells can spread to other tissues through blood circulation or lymphatic system [2]. Hence, cancer is very difficult to be treated.

Clinical diagnostics, prognostics, and targeted therapeutics of cancer need across-the-board comprehending molecular mechanisms of cancer cells. One of the remaining challenges is to distinguish functional mutations vital for cancer development, which is so-called “driver mutations,” and filter out the unfunctional and random “passenger mutations” [3]. With the development of next-generation DNA sequencing technologies, large-scale cancer genomics projects have been implemented to help researchers to identify driver genes, driver mutations, and driver pathways which promote cancer proliferation in large numbers of cancer patients [4–6]. Hence, it is necessary to find efficient methods for identifying mutated driver pathways in cancer cells, which can be further used to aid in designing effective drugs to treat cancer [7, 8].

In the past years, in gene level, several studies have been devoted to predict driver mutation with significantly higher mutation rate than background mutation rate in a large cohort of cancer patients. These methods have detected several gene mutations in cancer progression. However, even cancer genomes from the same type of cancer, no two genomes exhibit exactly the same complement of somatic aberrations. In other words, these approaches cannot capture the heterogeneity of genome mutations [9, 10].

As it is well known, same pathway may result from different genome aberrations [11, 12]. Hence, it is significant to study gene in pathway level, rather than in gene level. In pathway level, it is easy to capture the heterogeneous phenomenon in cancer cells [13, 14]. Until now, most of the studies analyze known pathway for enrichment of somatic mutations [9, 10, 15]. Though several pathways find out significantly perturbed genes [16–18], unfortunately, knowledge of pathways remains incomplete, and many pathway databases contain overlap and unavailable data. Therefore, taking into account these obvious limitations, it is indispensable to develop de novo discovery of mutated driver pathways without relying on prior knowledge.

In the whole genome, there are a huge number of gene sets if testing exhaustively. For instance, there are more than 10^26^ sets of seven human genes [19]. Therefore, testing all the groups up to a reasonable size seems implausible. However, in recent years, several studies have provided some methods to solve this problem [12, 20]. In these studies, the researchers find that there are two constraints on combinatorial patterns of mutations in cancer. First, generally, a driver mutation is rare. Particularly, researchers find that a single mutation is frequently enough to perturb one way. In other words, there is a phenomenon of mutual exclusivity between driver mutations. Second, a significant cancer pathway should cover a great majority of patients. Thus, the mutations should be contained by most patients in the pathway. This property is called high coverage. Lately, based on these two constraints, Vandin et al. [19] proposed a new and effective method, which defined a novel scoring function using the above two properties to detect the mutated driver pathway using the cancer data detected by next-generation DNA sequencing technologies. They defined the maximization of this method as the maximum weight submatrix problem. However, this problem is computationally difficult to solve.

In order to solve this problem, in this paper, based on GA method introduced by Zhao et al. [21], we propose the simulated annealing hybrid genetic algorithm (SAGA) method for mutated driver pathway detecting. In particular, we incorporate the gene expression data to improve GA to detect mutated driver pathway, and the experimental results on both simulated and real data show that the proposed method is effective.

The rest of this paper is organized as follows. In Section 2, some materials and methods used throughout this paper are introduced. Then, in Section 3, to test the efficiency of our methods, we apply our methods onto simulated data and two biological datasets. The results show that our methods are more efficient. Finally, we draw our conclusions in Section 4.

## 2. Materials and Methods

### 2.1. A Brief Introduction

**Figure 1:**
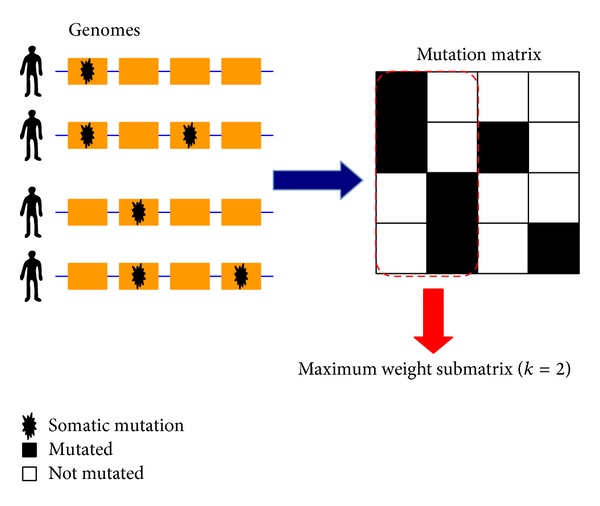
Somatic mutations in samples (patients) are represented in a mutation matrix.

Identifying driver pathway is extremely difficult. Considering this point, some researchers transformed this problem into maximum weight submatrix problem using two criteria [19], that is, “high coverage” and “high exclusivity.” However, this problem is NP-hard. In other words, no algorithm efficient in every case awaits a satisfactory result. Hence, many researchers use stochastic search methods to solve this problem. Particularly, Vandin et al. [19] proposed a method using these two properties (Figure 1). The first one is “high coverage,” which means the majority of samples have at least one mutation in driver pathway; the second one is “high exclusivity,” which means that lots of samples have no more than one mutation in one driver pathway. They reflect these two properties using a mutation matrix and a scoring function. A binary mutation matrix *A* is constructed by *m* rows (samples) and *n* columns (genes). The maximum weight submatrix problem is defined as selecting a submatrix *M* of size *m* × *k* in the mutation matrix *A* by calculating maximizing the scoring function:

(1) $$\begin{array}{c} W\left(M\right)=\left|\Gamma \left(M\right)\right|-\omega \left(M\right)=2\left|\Gamma \left(M\right)\right|-\sum_{g\in M}\left|\Gamma \left(g\right)\right|, \end{array}$$

where Γ(*g*) = {*i* : *A*_*ig*_ = 1} denotes that gene *g* in *i*th row (sample) is mutated. Γ(*M*) = ⋃_*g*∈*M*_Γ(*g*) represents the set of patients, in which at least one of the genes in *M* is aberrations. So, |Γ(*M*)| indicates the coverage of *M*· *ω*(*M*) = ∑_*g*∈*M*_|Γ(*g*)| − |Γ(*M*)| denotes the coverage overlap weight. In order to solve this problem, Vandin et al. [19] proposed the Markov chain Monte Carlo (MCMC) method. After that, Zhao et al. [21] used the genetic algorithm (GA) to solve this problem and achieved good experimental results. However, to avoid tripping in a local solution, local search method proposed by them is not good enough to solve this problem.

### 2.2. Simulated Annealing Hybrid Genetic Algorithm (SAGA)

As Zhao et al. [21] discussed, the genetic algorithm (GA) is a stochastic and powerful technique that can be effective in solving the maximum weight submatrix problem. However, there is a phenomenon called “premature” that maybe appear in the optimal solutions of GA. In other words, the result may be trapped in a local solution. Taking into account this situation, in this paper, we propose to use simulated annealing hybrid genetic algorithm (SAGA) to solve this problem. Simulated annealing (SA) as an optimization and heuristic algorithm mimics certain thermodynamic principles of producing an ideal crystal, which solve large-scale optimization problems in order to achieve a global optimal solution [22]. SA has been widely used in operational research problems. For example, Chu et al. [23] used SA to analyze the network of interacting genes. They detected the genes which control embryonic development and other biological processes. The details of our implementation, named simulated annealing hybrid genetic algorithm (SAGA), for the maximum weight submatrix problem based on SA are described as follows.

**Step 1**

Initialize the temperature *S*_0_.

**Step 2**

Use GA method to generate initial solution submatrix *M*, and generate the scoring function *W*(*M*).

**Step 3**

Using GA method to generate a new solution submatrix *M*′, in the neighborhood of current solution *X*, reevaluate the scoring function *W*(*M*′).

**Step 4**

If the generated solution submatrix scoring *W*(*M*′) is larger than former *W*(*M*), put *M* = *M*′. Update the existing optimal solution and go to Step 6.

**Step 5**

Else accepts *M*′ with probability

(2) $$\begin{array}{c} p=e^{\Delta S/T}, \end{array}$$

where

(3) $$\begin{array}{c} \Delta S=W\left(M^{\prime }\right)-W\left(M\right). \end{array}$$

If the solution is accepted, then *M* = *M*′. Update the existing optimal solution.

**Step 6**

Decrease the temperature periodically.

**Step 7**

Repeat Step 2 through 6 until stopping criterion is met.

**Figure 2:**
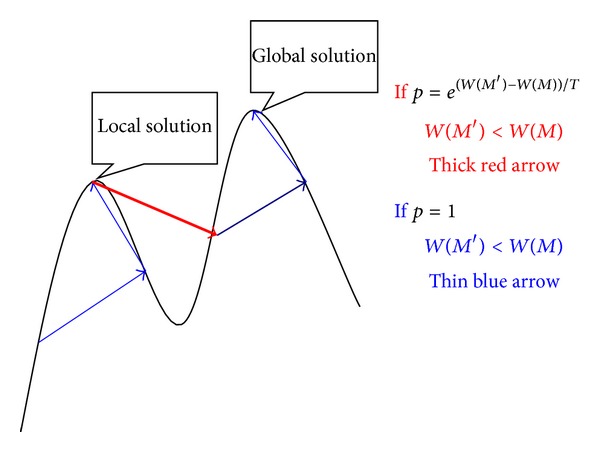
Simulated annealing: escape from local maximum solution.

Figure 2 shows the process of SA. It can be seen clearly that we can solve the global maximum solution by using SA.

### 2.3. Integrating with Gene Expression Data

**Figure 3:**
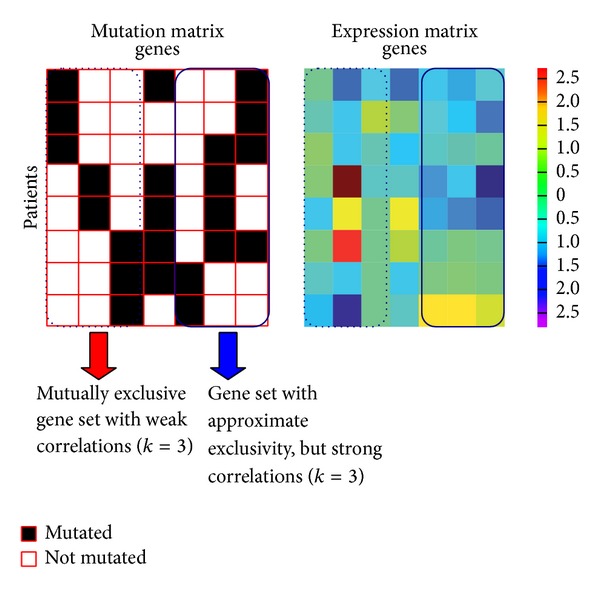
Illustration of the advantage of the integrative model. It utilizes the phenomenon that the expression profiles of gene pairs in same pathway have stronger correlations than those in different pathways to detect the driver mutation pathways. In blue dashed box, the genes have very weak expression correlations between each other, while, in the blue real line box, the genes with approximate exclusivity are strongly correlated with each other.

In biology, generally, there is noise and/or other factors contained in the data. On the other hand, multiple optimal solutions maybe occur. Taking into account this situation, Zhao et al. [21] proposed a new method called integrative model to deal with this problem. Their new method is based on a phenomenon: the expression profiles of gene pairs in same pathway have stronger correlations than that in different pathways (Figure 3). Hence, they combine the mutation submatrix and the gene expression data, which can distinguish the same score for selecting mutation pathway. They define the integrative model function as follows:

(4) FME=W(M)+λ∗R(EM),

where *R*(*E*_*M*_) = ∑_*j*1≠*j*2_(|pcc(*x*_*j*1_, *x*_*j*2_)|/(*k*(*k* − 1)/2)),  *E*_*M*_ is the gene expression submatrix which corresponds to the same gene set with the mutation submatrix *M*, and pcc(·) is the Pearson correlation coefficient. *x*_*j*1_ and *x*_*j*2_ are the expression data, which correspond to *j*_1_ and *j*_2_ in *E*_*M*_. Therefore, *R*(*E*_*M*_) is an additional term which enhances the biological correlation. *λ* is a coefficient. When *λ* = 1, *F*_ME_ will distinguish driver mutation from the same *W*(*M*). When *λ* ≥ 1, *F*_ME_ will detect the gene set with high correlation and exclusivity. In our study, we set *λ* = 1 and *λ* = 10. We apply SAGA into the integrative model, and it is more efficient compared with GA method for solving the maximum weight submatrix problem.

## 3. Results

We first tested the ability of the SAGA to detect the set *M* of maximum weight submatrix and compared the result with the MCMC and GA methods.

### 3.1. Simulation Study

We adopt the method represented by Zhao et al. [21]. The details of their implementation of simulated mutation data start with five gene sets *M*_1_, *M*_2_,…, *M*_*k*_. Each set has *k* members (*k* = 5 has been used in this study). For each row, we set the number to 1 (chosen uniformly at random) in *M*_*i*_  (*i* = 1,2,…, 5) with probability *p*_*i*_  (*p*_*i*_ = 1 − *i* · Δ, Δ = 0.05 has been used in this study), and if gene is 1 already, after that, with probability *p*_0_ we set the others to 1 in *M*_*i*_ (*p*_0_ = 0.04 has been used in this study). We can see that *p*_*i*_ indicates the coverage of *M*_*i*_ and *p*_0_ indicates exclusivity of *M*_*i*_. The others in *M*_*i*_ are mutated using a random model based on the observed characteristics of the glioblastoma data. This is the background mutation rate in *M*.

**Figure 4:**
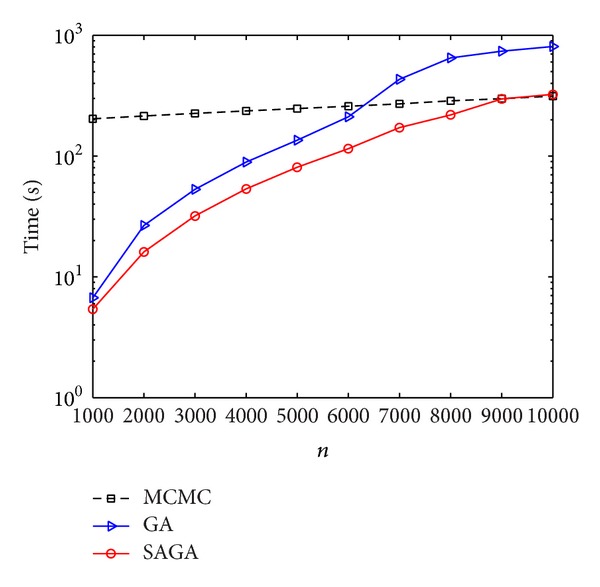
Comparison of computational time of SAGA, GA, and MCMC in terms of gene number from 1000 to 10000. In this plot, we use semilog coordinate (the *y*-axis) to show the computational time in seconds. All the makers correspond to the results of an average over 10 times.

We have compared the time complexity of MCMC, GA, and SAGA on selecting the submatrix of maximum weight (Figure 4). From this picture, we can see clearly that the GA is faster than MCMC when *n* is less than about 5000. Particularly, SAGA is always faster than GA from *n* = 1000 to *n* = 10000. In fact, it is well know that, for almost all of real applications, the *n* is smaller than 5000. On the other hand, the results of SAGA are the same as GA method; that is, they can both detect the five pathways.

Then we use an exact approach to test the accuracy of these methods, which is called binary linear programming (BLP) model proposed by Zhao et al. [21]. We run the BLP method to compare MCMC and GA performance with SAGA. After processing the data, the accuracy of GA and SAGA is equal, which is 95%, but higher than that of MCMC, which is 44%. In summary, our SAGA method has competitive efficiency with GA and MCMC.

### 3.2. Biological Applications

In this subsection, we applied our SAGA method onto lung adenocarcinoma and glioblastoma datasets. It needs to be emphasized that we consider the mutations in the same samples as one “metagene.” We use this criterion when we solve the maximum weight submatrix problem for further analysis. Using the same methods as Zhao et al. [21], we adopt the permutation test to assess the significance of the identified gene patterns. Not only do we get “best” results, but also we check the second optimal patterns, which move the “optimal” submatrix and then detect the “optimal” results in the new matrix.

We first apply our SAGA method onto lung adenocarcinoma. Comparing with GA method, we found that both of them can get the exact same “optimal” submatrix. However, the time using our method is less than that of GA. Afterwards, we apply SAGA-integrative model onto mutation matrix and gene expression matrix of glioblastoma. Compared with the integrating method in Zhao et al., like the former experiment, our method has the same results but using less time.

#### 3.2.1. Lung Adenocarcinoma

**Figure 5:**
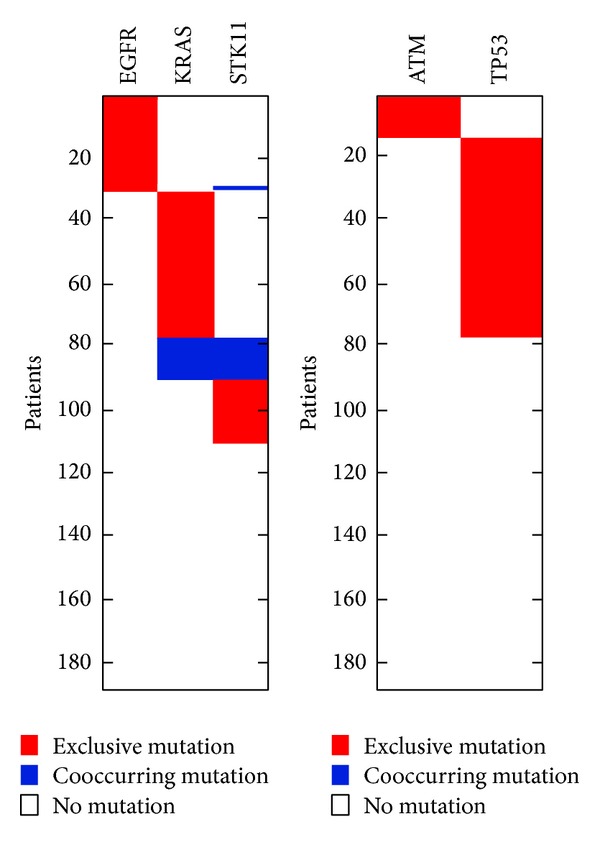
The high weight submatrices of the “optimal” gene set in the lung adenocarcinoma data. In this picture: red, exclusive mutation; blue, cooccurring mutation; white, no mutation. It is similar to Figure 7.

We applied our SAGA method to analyzing a dataset of 1013 somatic mutations identified in 188 lung adenocarcinoma patients' 623 sequenced genes from the Tumor Sequencing Project [9]. According to statistics, there are 365 genes mutated in at least one patient. We run the SAGA for sets of size 2 ≤ *k* ≤ 10. After running this algorithm, when *k* = 2, the pair EGFR and KBAS is the maximum weight submatrix. When *k* = 3, the most significant triplet is EGFR, KRAS, and STK11. When *k* ≥ 4, all sets are sampled with frequency < 0.3%. Then we perform a permutation test, as described in Vandin et al. [19]. The *P* value obtained is 0.018, which is larger than that of the triplet (EGFR, KRAS, and STK11). In other words, the triplet (EGFR, KRAS, and STK11) is at least as significant as the pair (EGFR and KRAS). In biology, we find that EGFR, KRAS, and STK11 are all involved in the pathway of mTOR (Figure 5). In Ding et al. [9], the mTOR pathway is very important for lung adenocarcinoma. Hence, our method can seek out driver pathway.

**Figure 6:**
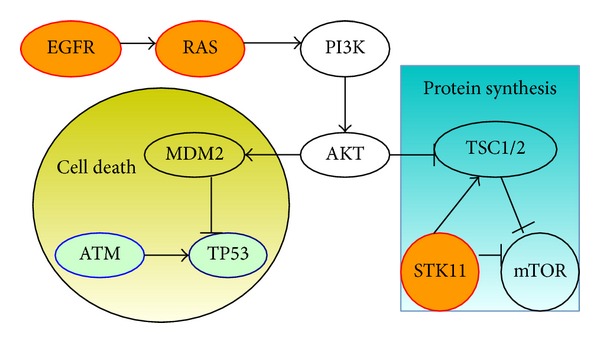
In mTOR signaling pathway, there is the triplet of genes codes for proteins (orange nodes), and the pair (ATM and TP53) corresponds to interacting proteins in the cell cycle pathway (light blue nodes). These two pathways are reported in Ding et al. [9].

We remove the above three genes and apply the method to detect the additional gene sets. On the remaining genes, when *k* = 2, we identify the gene set (ATM, TP53) that is mutated with frequency 56% and find that the weight of the pair is significant (*P* < 0.01). Previous studies have shown that both ATM and TP53 are in the cell cycle checkpoint control and direct interaction [24, 25] (Figure 6).

#### 3.2.2. Glioblastoma

We next analyzed a collection of somatic single-nucleotide mutations and gene expression profiles identified from 206 glioblastoma multiforme samples from The Cancer Genome Atlas [15]. After processing these data, we established two matrices, that is, a mutation matrix and an expression matrix, which cover 90 samples and 1126 genes.

Firstly, we discover the mutation pattern only depending on the mutation matrix. When *k* = 2, we identify gene pairs (CDKN2A and TP53), and the other is CDK2B and one “metagene” containing TSPAN31 and CDK4. However, using previous methods to solve the original maximum weight submatrix problem, we cannot solve this problem, because there are two same score “optimal” gene sets. Then, we apply integration model onto these “optimal gene sets.” Running the process of integrative method with mutation matrix and gene expression matrix, we find that the correlation between CDK4 and CDKN2B has high score compared to that between TSPAN31 and CDK4. In other words, CDK4 is stronger correlation than TSPAN31 with CDK2B. In biological research, we find that the genes CDK4, CDKN2B are part of RB signaling pathway; however, there is no evidence to discover the relation between TSPAN31 and CDKN2B. In another point of view, it proves the advantages of integrative method. When *k* = 3, the optimal solution is CDK4, CDKN2B, and RB1. After that, we perform a permutation test, as described in Vandin et al. [19]. We find that the triplet (RB1, CDKN2B, and CDK4) is more significant than the pair (CDK4 and CDKN2B).

**Figure 7:**
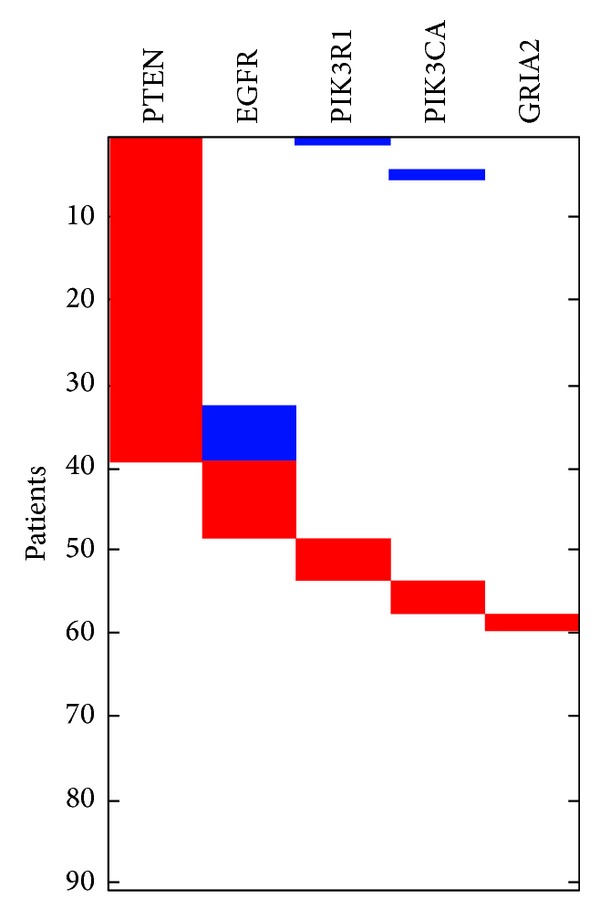
The high weight submatrix of “optimal” gene set after moving the sets (PTEN, EGFR, PIK3R1, PIK3CA, and GRIA2) in glioblastoma data.

**Figure 8:**
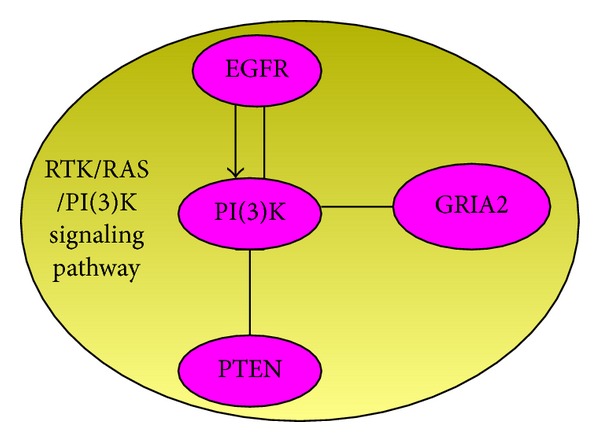
The genes sets (PTEN, EGFR, PIK3R1, and PIK3CA) are involved in the RTK/RAS/PI(3)K signaling pathway, which is reported in TCGA [15].

We remove these five genes (RB1, CDKN2A, CDKN2B, CDK4, and TP53) from the mutation matrix and then apply SAGA to discover the others genes. When *k* = 5, the optimal result is PTEN, EGFR, PIK3R1, PIK3CA, and GRIA2, which is significant in the other solutions (Figure 7). The set (PTEN, EGFR, PIK3R1, and PIK3CA) is all part of RTK/RAS/PI(3)K signaling pathway (Figure 8). In biology, gene GIRA2 is very important in glioma cells [26, 27].

## 4. Discussion and Conclusion

In bioinformatics, it is important to detect mutated driver pathway in cancer cells. In this paper, we introduce an algorithm for discovering mutated driver patterns de novo using somatic mutation data from biological datasets, which is based on recent exploration made by Vandin et al. [19] and Zhao et al. [21]. We proposed an optimization and heuristic algorithm, that is, simulated annealing hybrid genetic algorithm, which is named SAGA. By means of simulation study, we proved that our SAGA method had completive efficiency with GA and MCMC. Then, we applied our method onto lung adenocarcinoma and glioblastoma. Particularly, we considered incorporating the gene expression data into SAGA method to improve its performance, which achieved satisfactory results. Not only are the results the same as GA, but also the arithmetic speed of SAGA is faster than that of GA.

Although the proposed method can find mutated driver pathway without relying on prior knowledge, we should note that the assumption of high exclusivity and high coverage is too strict for selecting the driver pathway. In biological application, mutual exclusivity is a fairly strong assumption, which holds only for driver mutations in the same pathway. It is well known that driver mutations may be caused by multiple pathways, such as cooccurring and possibly cooperative. For example, acute myeloid leukemia is caused by CBF translocations and kinase mutations [28]. So, we emphasize that assumption of mutual exclusivity occurs only in the same driver pathway. In the future, we will study the biological data, such as DNA methylation and copy-number variant (CNV), exploring the regular pattern of cooccurring and the other mutated driver pathways.
